# Comment on Teles et al. HIIE Protocols Promote Better Acute Effects on Blood Glucose and Pressure Control in People with Type 2 Diabetes than Continuous Exercise. *Int. J. Environ. Res. Public Health* 2022, *19*, 2601

**DOI:** 10.3390/ijerph19138028

**Published:** 2022-06-30

**Authors:** Victor Hugo Gasparini Neto, Leticia Nascimento Santos Neves

**Affiliations:** Center of Physical Education and Sports, Laboratory of Exercise Physiology (LAFEX), Federal University of Espírito Santo, Vitoria 29075-910, Brazil; leticiansn@gmail.com

After a careful appraisal, we are concerned that the article “HIIE Protocols Promote Better Acute Effects on Blood Glucose and Pressure Control in People with Type 2 Diabetes than Continuous Exercise” [1] may have some errors that warrant further review by the editor and authors, and which may impact the original article’s conclusions.

**Point 1**

Regarding the reported statistical description: The article did not note if a normality test was conducted. Additionally, in the supplementary files, the authors highlight those seven variables passed, but eight did not pass in normality. It seems that the authors chose the Kolmogorov–Smirnov test to assume normality, and this test is used with samples up to 100, but the Shapiro–Wilk test is preferred for samples less than 50 [2]. If the assumption of normality is violated, interpretation and inference may not be reliable or valid [2]. More than 50% of the variables do not pass in normality test. The RM ANOVA criteria were violated, and the authors indicate the use of One-Way ANOVA in the results (this is conflicting information). Version 2.0 of SPSS does not exist. The eta squared does not have a reference for interpretation.

**Point 2**

The entire article needs major revisions regarding terminology. Including the following: The maximal oxygen consumption ($\overset{\cdot }{V}$O_2max_) and $\overset{\cdot }{V}$O_2peak_ were not the same terminology and did not present a standard. The medication Losartan was written incorrectly. Glycated hemoglobin (HbA1c) was written incorrectly in various sentences. The RPE (rate of perceived exertion) was described in the methods, and SPE was described in the results (Table 2). Five participants initiated the exercise with blood glucose higher than 250 mg/dL, which is not recommended [3]. The blood glucose data for subject number seven available on Google Drive present a value of 1110 at peak value. This value is wrong, because the Accu-Chek Performa glucometer indicates a maximal value of 600 mg/dL. Different blood pressure monitor types and models were used: an oscillometric sphygmomanometer (OMRON HEM-705 described at data collection and OMROM HEM-7122) and a mercury sphygmomanometer (auscultatory), which violates the internal consistency. It is not clear which arm was measured—both, right, or left arm? It is essential to describe this information according to the guidelines: “measure BP in both arms, preferably simultaneously. If there is a consistent difference between arms > 10 mmHg in repeated measurements, use the arm with the higher BP” [4].

Another critical point is the incremental test protocol. “The test started with a two-minute warm-up, and then the speed was increased by 0.1 km/h every 10, 20, or 30 s until exhaustion, without inclination”. How did this increment work? It is not clear. It is necessary to insert the bibliography to determine this protocol. We suggest the authors insert a reference for the protocol. Since 1996, a 1% treadmill grade most accurately reflects the energetic cost of outdoor running [5].

**Point 3**

In the discussion section, the authors cited the Santiago et al. (2017) study, which demonstrated reductions in BP and blood glucose after continuous and interval exercise [6]. However, the Santiago study did not cite or analyze glycolytic and oxidative enzymes, as mentioned in the present article: “In addition, there was an increase in the activity of glycolytic and oxidative enzymes” [1].

**Point 4**

In conclusion, we flag concerns about the data extraction accuracy, its analysis, and procedures that cannot be replicable (one principle of good and clear science). We, therefore, respectfully seek clarification and major revision.
